# Assessment of High School Students’ Participation in Blood Donation and Registration as an Organ Donor

**DOI:** 10.1001/jamanetworkopen.2020.16377

**Published:** 2020-09-18

**Authors:** John Tat, Barton Hays, Martin Teachworth, Alexander Kuo, Renate B. Pilz, Beatrice A. Golomb, Gerry R. Boss

**Affiliations:** 1 Department of Medicine, University of California, San Diego, La Jolla; 2 San Diego Science Educators Association, San Diego, California; 3 Comprehensive Transplant Center, Cedars-Sinai Medical Center, Los Angeles, California

## Abstract

This cross-sectional study assesses the association between blood donation and willingness to register as an organ donor among California high school students.

## Introduction

Organ donor availability limits organ transplantation. Strategies are needed to increase organ donation, especially among minority populations.^1^ Altruism motivates blood and organ donors. Thus, blood donors might be potential targets to increase organ donation.

We assessed organ donor registration rates of high school students, comparing blood donors with non–blood donors. In California, anyone age 17 years or older can donate blood, and those 13 years or older can register as organ donors.

## Methods

This study was conducted in 4 high schools from geographically and socioeconomically distinct areas in California with diverse racial/ethnic student populations. Participating students completed a questionnaire administered by student surveyors trained by 2 science teachers (B.H. and M.T.) and a university professor (A.K.) (eAppendix in the Supplement). Student surveyors were instructed to approach students randomly; of those approached, approximately 80% of students agreed to participate. This cross-sectional study was approved by the University of California, San Diego, institutional review board. No personally identifiable data were collected, so informed consent was waived.

Analyses were performed using the Fisher exact test and multivariate logistic regression, and reporting followed the Strengthening the Reporting of Observational Studies in Epidemiology (STROBE) guideline for cross-sectional studies. The threshold for statistical significance was set at 2-sided *P* = .05. More information about the methods used in the study is provided in the eAppendix in the Supplement.

## Results

We surveyed 1784 students: 814 (45.6%) male students, 784 (43.9%) Hispanic, 482 (27.0%) White, 180 (10.1%) African American, 151 (8.5%) multiracial, 142 (8.0%) Asian, and 17 (1.0%) American Indian (Table 1). Among 953 blood donors, 314 students (32.9%) were registered as organ donors, compared with 198 of 831 non–blood donors (23.8%) (*P* < .001) (Table 1). Significance was maintained when analyzed by sex: 144 male (35.1%) and 170 female (31.3%) blood donors were registered as organ donors, compared with 95 male (23.5%) and 103 female (24.1%) non–blood donors (*P* < .001 for male students, and *P* = .01 for female students) (Table 1). When analyzed by race/ethnicity, more White blood donors were registered as organ donors than White non–blood donors (118 blood donors [47.2%] vs 58 non–blood donors [25.0%]; *P* < .001) (Table 1). There were no other statistically significant differences by race/ethnicity.

**Table 1.** Demographic Characteristics and Blood and Organ Donor Status Stratified by Blood Donor Status

**Characteristic**^a^	Total, No. (%)	Blood donor status, No. (%)		Organ donor status, No. (%)		Registered as an organ donor^b^			Willing to register as an organ donor		
		Blood donor	Non–blood donor	Registered	Not registered	Blood donor, No. (%)	Non–blood donor, No. (%)	*P* value^c^	Blood donor, No. (%)	Non–blood donor, No. (%)	*P* value^c^
All	1784	953	831	512	1272	314 (32.9)	198 (23.8)	<.001	367 (57.4)	332 (52.5)	.08
Male students	814 (45.6)	410 (50.4)	404 (49.6)	239 (29.4)	575 (70.6)	144 (35.1)	95 (23.5)	<.001	115 (43.2)	158 (51.1)	.07
Female students	970 (54.4)	543 (56.0)	427 (44.0)	273 (28.1)	697 (71.9)	170 (31.3)	103 (24.1)	.01	252 (67.6)	174 (53.7)	<.001
Race/ethnicity											
African American	180 (10.1)	87 (48.3)	93 (51.7)	50 (27.8)	130 (72.2)	28 (32.2)	22 (23.7)	.24	37 (62.7)	46 (64.8)	.86
American Indian	17 (1.0)	7 (41.2)	10 (58.8)	5 (29.4)	12 (70.6)	4 (57.1)	1 (10.0)	.10	1 (33.1)	3 (33.1)	>.99
Asian	142 (8.0)	62 (43.7)	80 (56.3)	35 (24.6)	107 (75.3)	18 (29.0)	17 (21.3)	.33	21 (47.7)	27 (42.9)	.69
Hispanic	784 (43.9)	445 (56.8)	339 (43.2)	201 (25.6)	583 (74.4)	116 (26.1)	85 (25.1)	.80	185 (56.2)	144 (56.7)	.93
Multiracial	151 (8.5)	87 (57.6)	64 (42.4)	40 (26.5)	111 (73.5)	27 (31.0)	13 (20.3)	.19	35 (58.3)	27 (52.9)	.70
White	482 (27.0)	250 (51.9)	232 (48.1)	176 (36.5)	306 (63.5)	118 (47.2)	58 (25.0)	<.001	82 (62.1)	81 (46.6)	.008

^a^ Of the 1784 participants, 28 did not provide their racial/ethnic background, and hence the race/ethnicity subgroups add up to 1756 participants.

^b^ Organ donor registration status across races was significantly different (*P* < .001).

^c^ *P* is the probability value for the comparison between blood donors and non–blood donors.

Among students not already registered as organ donors, more than half were willing to register as organ donors, 367 blood donors (57.4%) and 332 non–blood donors (52.5%) (Table 1). There was no statistically significant difference between blood donors and non–blood donors, but more female blood donors were willing to register as organ donors than female non–blood donors (252 blood donors [67.6%] vs 174 non–blood donors [53.7%]; *P* < .001); there was no difference for male students. Comparing by sex, more female blood donors were willing to register as organ donors than male blood donors (252 female students [67.6%] vs 115 male students [43.2%]; *P* < .001); no difference existed between male and female non–blood donors. Among racial/ethnic groups, more White blood donors were willing to register as organ donors than White non–blood donors (82 blood donors [62.1%] vs 81 non–blood donors [46.6%]; *P* = .008). This was not true for other racial/ethnic groups (eg, non-White Hispanic: 185 blood donors [56.2%] vs 144 non–blood donors [56.7%]; *P* = .93).

Multivariable regressions qualitatively affirmed these findings (Table 2). Blood donors were significantly more likely to be registered organ donors (odds ratio [OR], 1.60; 95% CI, 1.29-1.97; *P* < .001). Interactions of blood donor status with organ donor registration were significant for race/ethnicity (eg, White race: OR, 2.12; 95% CI, 1.34-3.36; *P* < .001), and sex (eg, organ donation willingness for male blood donors: OR, 0.40; 95% CI, 0.26-0.64). This supported stratified analyses, which corroborated the finding that blood donation was associated with organ donor registration in White students (OR, 2.73; 95% CI, 1.85-4.04; *P* < .001), and to organ donation willingness in female students (OR, 1.79; 95% CI, 1.32-2.44; *P* < .001).

**Table 2.** Multivariate Analysis of Organ Donor Registration Status and Willingness to Donate Organs

Factor	SE	OR (95% CI)	*P* value
**Organ donor registration status (n = 1784)**			
Limited model			
Blood donor	0.17	1.60 (1.29-1.97)	<.001
Male students	0.11	1.04 (0.84-1.28)	.71
White	0.19	1.67 (1.34-2.09)	<.001
Model with interaction term			
Blood donor	0.16	1.27 (0.99-1.63)	.06
Male students	0.11	1.05 (0.85-1.29)	.67
White	0.20	1.09 (0.76-1.55)	.65
Blood donor × White	0.50	2.12 (1.34-3.36)	.001
Among non-White participants (n = 1302)			
Blood donor	0.16	1.27 (0.98-1.63)	.07
Male students	0.13	0.97 (0.76-1.25)	.84
Among White participants (n = 482)			
Blood donor	0.55	2.73 (1.85-4.04)	<.001
Male students	0.24	1.23 (0.84-1.82)	.29
**Willingness to donate organs (n = 1272)**			
Limited model			
Blood donor	0.13	1.18 (0.94-1.47)	.15
Male students	0.07	0.58 (0.46-0.73)	<.001
White	0.13	0.98 (0.75-1.27)	.87
Model with interaction term			
Blood donor	0.28	1.79 (1.32-2.44)	<.001
Male students	0.14	0.91 (0.66-1.24)	.53
White	0.13	0.96 (0.74-1.25)	.77
Blood donor × male	0.09	0.40 (0.26-0.64)	<.001
Female students only (n = 697)			
Blood donor	0.28	1.79 (1.32-2.44)	<.001
White	0.18	0.94 (0.65-1.36)	.74
Male students only (n = 575)			
Blood donor	0.12	0.73 (0.52-1.01)	.06
White	0.19	0.98 (0.68-1.42)	.93

Abbreviation: OR, odds ratio.

## Discussion

This cross-sectional study found that high school student blood donors were registered as organ donors at higher rates than non–blood donors. White blood donors were more likely to be willing to register as organ donors than White non–blood donors, and there were no other statistically significant differences by race/ethnicity. Among those not registered as organ donors, female blood donors were more likely to be willing to register than non–blood donors.

If expressed willingness to register as an organ donor translates to registration, then simply asking high school students to register as organ donors might increase registration. However, intent does not always translate to action.^2,3^

This study had limitations. First, students self-reported registration and willingness to register as organ donors; prosocial behaviors can be overreported.^4,5^ Second, the questionnaire was purposefully short to prevent survey exhaustion, which precluded assessing factors that may motivate or deter organ donor registration. Third, most blood donors were surveyed immediately after donating blood, an altruistic activity, which could have led to overreporting of willingness to be organ donors.

## Notes

Supplement. **eAppendix.** Supplementary Methods and Survey Questionnaire **eReferences**
