# Supplementary material for: Assessment of High School Students’ Participation in Blood Donation and Registration as an Organ Donor
Source: JAMA Netw Open. 2020 Sep 18;3(9):e2016377. doi: 10.1001/jamanetworkopen.2020.16377 (PMC7501534; doi:10.1001/jamanetworkopen.2020.16377)
Supplement: Supplement. — eAppendix. Supplementary Methods and Survey Questionnaire eReferences [file jamanetwopen-e2016377-s001.pdf]

Supplementary Online Content

Tat J, Hays B, Teachworth M, et al. Assessment of high school students' participation in blood donation and registration as an organ donor. *JAMA Netw Open*. 2020;3(9):e2016377. doi:10.1001/jamanetworkopen.2020.16377

eAppendix. Supplementary Methods and Survey Questionnaire

eReferences

This supplementary material has been provided by the authors to give readers additional information about their work.

© 2020 Tat J et al. *JAMA Network Open.*

eAppendix. Supplementary Methods

Selection of schools

We selected three high schools in San Diego County and one in Riverside County based on: (i) geographically and socioeconomically distinct areas; (*ii*) large student bodies with high rates of non-White students; and (*iii*) willingness of the school administration to participate in the study. The schools and their percent of non-White students were: Helix Charter High School, La Mesa, CA, (81%), La Jolla High School, La Jolla, CA (46%), Lakeside High School, Lake Elsinore, CA (76%), and Oceanside High School, Oceanside, CA (79%).

Selection and training of study surveyors

At each school, we recruited about 20 student-surveyors annually from clubs or classes focused on healthcare careers. We asked the club advisor or the classroom teacher to recommend students, based on the student's communication and organization skills, reliability, and availability.

Selected students were taught by two science teachers (BH and MT) and a university professor (AK) about organ donation and how to conduct the survey. This training, which occurred during non-school hours, served as the academic component of a service-learning experience, and helped to assure consistency among the surveyors.

We used students as study surveyors for three reasons. First, in almost all California high schools, community service is a graduation requirement. Participating in the study as a student-surveyor qualified as service hours. Thus, students were motivated to serve as surveyors, providing the study with a reliable and sustainable source of surveyors. On average, each student-surveyor received ~25 community service hours per year. Second, we reasoned that surveyed students would be more receptive and honest with a fellow student-surveyor than an adult-surveyor. Students feel less pressured when discussing healthcare topics with peers, compared to a perceived authoritative figure, such as an adult, a teacher, or a university professor.[1-3] Thus, student-surveyors should reduce response bias. And third, we hoped exposure to the socio-medical sciences through service-learning would motivate student-surveyors to pursue science or math careers in the future, because exposure to math and science has been reported to positively impact a student's decision to declare these majors in college.[4,5]

Survey approach

The student surveyors asked other students if they were interested in participating in a study concerning organ donor registration. The student surveyors were specifically instructed to approach other students "randomly," and not to seek out only their friends. The percentage of

© 2020 Tat J et al. *JAMA Network Open.*

the student body that was approached varied between 4.4% at Helix Charter High School to 11.8% at Lakeside High School. Of those who were approached, ~80% agreed to participate, with the demographic breakdown of the participating students reflecting the racial/ethnic demographics of the schools.

Most blood donors were surveyed at a campus blood drive during the 15-minute mandatory observation period after donating blood. Other blood donors and all non-blood donors were surveyed during non-class times, such as study halls, lunch time, and after-school events at the schools. A blood donor was defined as a person who had ever donated blood, including during an ongoing blood drive, and a non-blood donor was defined as a person who had never donated blood at any time. We relied on convenience sampling. No willing participant was excluded.

Those who agreed to participate in the study were asked to complete a questionnaire that had been pre-loaded onto an iPad (the questionnaire is at the end of the methods). The questionnaire started with a request for general demographical information, and then proceeded to questions about blood donation status and organ donation registration. Student surveyors were available to answer technical questions about the survey, but they were specifically instructed not to influence participants in terms of questionnaire responses.

To ensure adherence to the study protocol, the student surveyors received ongoing supervision from a student overseer. The student overseer was selected and trained by the responsible teacher.

Data collection and statistical analyses

Data for the three academic years between 2015 and 2017 were collected and managed in Excel. This was a cross-sectional study, and follow-up of respondents did not occur. Descriptive statistics are provided. We did not perform *a priori* power analysis. Bivariable testing of relationships (X^2) was supplemented by multivariable assessment employing logistic regression with robust standard errors. Multivariable models evaluating organ donor registration status, and organ donation willingness (among those not already registered as organ donors) examined prediction by blood donor status, sex, and ethnicity and tested interaction terms between blood donor status and sex, as well as blood donor status and ethnicity. Significance of an interaction term supported analysis stratified on the interacting variable. Of the variables used in multivariable analyses, only race/ethnicity had missing values, and for this variable, values were 98.4% complete. Missing values were not imputed. Analyses were conducted using Stata® 8.0 (College Station, TX). Statistical significance was defined as two-sided $p < 0.05$.

© 2020 Tat J et al. *JAMA Network Open.*

Survey Questionnaire

1. Are you a student enrolled in a school at this time? ☐Yes ☐No

2. What is the name of that school? ______________________________

3. What is your current grade in that school? ______________________________

4. Name the city of your residence. ______________________________

5. What is your gender? ☐Male ☐Female

6. What is your age? ______________________________
If you are under 18, you can't register to donate organs from this site but please continue with the survey

7. Were you born in the United States or are you a citizen of the United States?
☐Yes ☐No

8. Enter your primary language if it is not English ______________________________

9. What is your race?
☐Asian and Pacific Islander ☐African-American
☐Hispanic ☐American Indian
☐White ☐Multiracial

10. Have you ever donated blood in the past or are you expecting to donate blood now? ☐Yes ☐No

11. Have you ever registered to donate your organs? ☐Yes ☐No
This registration could have occurred through the DMV or other organizations. Organs can only be donated if the donor has died.

© 2020 Tat J et al. *JAMA Network Open.*

eReferences

1. Hindmarch T, Allikmets S, Knights F. A Narrative Review of Undergraduate Peer-based Healthcare Ethics Teaching. *International Journal of Medical Education.* 2015;6:184.

2. Carr S, Brand G, Wei L, et al. “Helping Someone with A Skill Sharpens it in Your Own Mind”: A Mixed Method Study Exploring Health Professions Students Experiences of Peer Assisted Learning (PAL). *BMC Medical Education.* 2016;16(1):48.

3. de Menezes S, Premnath D. Near-peer Education: A Novel Teaching Program. *International Journal of Medical Education.* 2016;7:160.

4. Wang X. Why Students Choose STEM Majors: Motivation, High School Learning, and Postsecondary Context of Support. *American Educational Research Journal.* 2013;50(5):1081-1121.

5. Kitchen JA, Sonnert G, Sadler PM. The Impact of College- and University-run High School Summer Programs on Students’ End of High School STEM Career Aspirations. *Science Education.* 2018;102(3):529-547.

© 2020 Tat J et al. *JAMA Network Open.*
